# Development of Performance and ERPs in a Flanker Task in Children and Adolescents with Tourette Syndrome—A Follow-Up Study

**DOI:** 10.3389/fnins.2017.00305

**Published:** 2017-06-12

**Authors:** Heike Eichele, Tom Eichele, Lynn Marquardt, Steinunn Adolfsdottir, Kenneth Hugdahl, Lin Sørensen, Kerstin J. Plessen

**Affiliations:** ^1^Department of Biological and Medical Psychology, University of BergenBergen, Norway; ^2^K.G. Jebsen Centre for Research on Neuropsychiatric Disorders, University of BergenBergen, Norway; ^3^Section for Neurophysiology, Department of Neurology, Haukeland University HospitalBergen, Norway; ^4^Division of Psychiatry, Haukeland University HospitalBergen, Norway; ^5^Child and Adolescent Mental Health Center, Mental Health Services Capital RegionCopenhagen, Denmark; ^6^Department of Clinical Medicine, Faculty of Health and Medical Sciences, University of CopenhagenCopenhagen, Denmark

**Keywords:** Tourette syndrome, ADHD, children, adolescence, event-related potentials, performance monitoring, developmental trajectories, follow-up

## Abstract

**Background:** Tourette Syndrome (TS) is a neurodevelopmental disorder with childhood-onset, with a typical decline in tic severity, as well as an increasing ability to suppress tics in late childhood and adolescence. These processes develop in parallel with general improvement of self-regulatory abilities, and performance monitoring during this age-span. Hence, changes in performance monitoring over time might provide insight into the regulation of tics in children and adolescents with TS.

**Method:** We measured reaction time, reaction time variability, accuracy, and event-related potentials (ERP) in 17 children with TS, including 10 children with comorbid Attention-Deficit/Hyperactivity Disorder (ADHD), 24 children with ADHD, and 29 typically developing children, using a modified Eriksen Flanker task in two testing sessions administered on average 4.5 years apart. We then compared task performance, as well as ERP components across groups, and over time using regression models.

**Results:** Task performance improved in all groups with age, and behavioral differences between children with TS and controls diminished at second assessment, while differences between controls and children with ADHD largely persisted. In terms of ERP, the early P3 developed earlier in children with TS compared with controls at the first assessment, but trajectories converged with maturation. ERP component amplitudes correlated with worst-ever tic scores.

**Conclusions:** Merging trajectories between children with TS and controls are consistent with the development of compensatory self-regulation mechanisms during early adolescence, probably facilitating tic suppression, in contrast to children with ADHD. Correlations between ERP amplitudes and tic scores also support this notion.

## Introduction

Tourette Syndrome (TS) is a neurodevelopmental disorder with childhood onset, defined by the presence of multiple motor tics and at least one vocal tic over the period of 1 year or more (American Psychiatric Association, 1994). Tics typically emerge at around 6 years of age and often follow a developmental time-course in which tics increase in frequency and severity to a worst-ever period around age 8–12 years, and become increasingly controlled during adolescence (Bloch and Leckman, 2009). This typical course coincides with the development of self-regulatory control during childhood and adolescence (Davidson et al., 2006; Tau and Peterson, 2010) and the maturation of the frontal cortex (Gogtay et al., 2004).

Compensatory neuromodulatory alterations in brain function possibly evolve through the constant need to suppress tics (Eichele and Plessen, 2013), resulting in increased sustained attention to emerging tics (or urges) and control over motor output/efferents. This process is thought to involve activity in a functional network that includes the frontal cortices, basal ganglia, and the thalamic nuclei, the so-called cortico-striato-thalamo-cortical circuits (Jackson et al., 2015). Activity in this network is also elicited during tasks requiring cognitive control and performance monitoring such as the Eriksen Flanker task (Eriksen and Eriksen, 1974). A variant of this task is used here in combination with event-related potentials (ERP) to identify underlying electrophysiological markers of attention and inhibitory control, which we assume is employed continuously to suppress tics. While other tasks have previously been used in EEG studies of children with TS, flanker tasks have only been used in behavioral studies in this patient group (Ozonoff et al., 1998; Crawford et al., 2005). We chose this task for our study as it is particularly useful for testing response inhibition and the ability to suppress and overwrite a pre-potent conflicting and opposing response that is prepared but incorrect, while the task is overall balanced for trial probability, and all trials require a motor response.

Several ERP components allow for a more detailed mapping of the performance monitoring process, beyond the importance to measure performance on the task. The P3 component is elicited by salient stimuli around 300 ms, and its dynamics can be described with predictive coding (Eichele et al., 2005). While its earlier subcomponents correspond to orienting and novelty, the later aspect of P3 is thought to more closely represent working memory and response selection (Donchin, 1981; Donchin and Coles, 1998; Polich, 2007), and is also sensitive to changes in cognitive conflict and control (Clayson and Larson, 2011a,b). After errors, the error-related negativity (ERN), as an immediate marker for automatic error detection and the subsequent error positivity (Pe), emerging at 300 ms after incorrect responses, associated with evaluation, awareness, and salience of errors are detectable. Both components show reduced amplitudes in neuropsychiatric disorders, including ADHD (Johnstone et al., 2013). Even though deficits in error processing have been studied extensively in children with ADHD (Johnstone et al., 2013; Plessen et al., 2016), there are to date only few available reports on ERN/Pe to visual tasks in children with TS (Shephard et al., 2016a,b), thus far showing no clear differences between individuals with uncomplicated/TS only and healthy controls. However, young people with TS and comorbid ADHD showed reduced amplitudes of ERN, Pe, and P3 were seen in a Go/Nogo task, while the presence of comorbidity in TS yielded no differences in the acquisition phase, and marginal differences in the reversal phase of a reinforcement learning experiment.

Most studies measuring performance monitoring in children with neuropsychiatric disorders have used a cross-sectional design. It is, however, difficult to study developmental aspects of behavior with cross-sectional methods, due to cohort effects or possible bias in the selection of different age-groups of participants (Kraemer et al., 2000). Moreover, the interpretation of cross-sectional ERP component amplitudes is challenging in children, due to the uncertainty, whether correlations with age represent physiological changes accompanying maturation or rather increments of cognitive abilities.

The general aim of this study was therefore to track performance monitoring and adaptive effects in children with Tourette syndrome over time. As many children with TS have comorbid ADHD, a contrast group with children with ADHD was included to leverage the impact of comorbid ADHD in combination with TS, as well as a control group. We examined all three groups clinically, behaviorally and with ERPs at two time points, on average 4.5 years apart in a longitudinal design, to avoid the shortcomings of cross-sectional designs. At the first assessment, we found that children with TS showed higher amplitudes of an early P3 component of the stimulus-locked ERPs in the grand average across experimental conditions and in separate trial outcomes. In the corresponding response-locked ERP data, children with TS had a slightly higher positive complex before the motor response, likely reflecting a late P3. Groups did not differ in post-response components. We assumed from those findings that children with TS employ additional attentional resources during stimulus evaluation as a compensatory mechanism to maintain performance (Eichele et al., 2016). We here re-examined the children at a second assessment, and focused on the developmental trajectories of the stimulus-locked attention-related potentials reflected by the early P3 and late P3, as well as on response-locked potentials related to error processing reflected by the ERN and the Pe.

Based on brain maturation and attenuation of tic symptoms during adolescence, as well as previous findings, we expected that children with TS would over time show (i) performance-monitoring pattern in terms of reaction times, variability of reaction times, and response accuracy similar to those of control children, whereas we expected better performance compared to children with ADHD. Further, we expected that children with TS would show (ii) ERP amplitudes similar to those of control children, whereas we expected that children with ADHD would show reduced ERP amplitudes in line with earlier findings. In addition, we explored associations between tic scores and ERP amplitudes under the assumption that tic control during adolescence is related to adaptation in control systems.

## Materials and methods

### Participants

The original participant group at the first assessment (T1) included 39 children with ADHD, 25 children with Tourette syndrome and 35 control children aged 8–12. The study was approved by the Regional Committee for Medical Research Ethics, West-Norway and written, informed consent was obtained from the legal guardians/parents of all non-adult research participants. Inclusion criteria and results relating only to this cohort are described elsewhere (Eichele et al., 2016). All children that initially participated at T1 were contacted again, and 70 children and adolescents with an age range 11–17 years participated in a follow-up ERP investigation after ~4.5 years [24 ADHD, 17 TS (7 TS “only,” 10 TS+ADHD), 29 controls], hence 70 participants attending both T1 and T2 were included here in the longitudinal design. Dropout rate was 29% for the overall group (17% controls, 38% ADHD, 32%TS). The follow-up investigation at T2 consisted of a semi-structured interview, the K-SADS (Schedule for Affective Disorders and Schizophrenia for School-Aged Children; Kaufman et al., 1997), the Children Global Assessment Scale (CGAS; Shaffer et al., 1983), and the DuPaul ADHD-Rating Scale (ADHD-RS; DuPaul et al., 1998), along with a best estimate consensus procedure that considered all available study material (Leckman et al., 1982). Children met diagnostic criteria for TS and ADHD, respectively (DSM-IV; American Psychiatric Association, 1994). Tic symptoms were assessed using the Yale Global Tic Severity Scale (YGTSS) yielding Total Motor (0–25), Total Phonic (0–25), and the combined Total Tic Score (0–50) for current and worst-ever tic severity separately in an interview with the child and parents (Leckman et al., 1989). At T1, exclusion criteria for the control group were a lifetime history of Tic disorder, OCD, ADHD, or a current DSM-IV axis I disorder other than specific (simple) phobias. Additional exclusion criteria for all groups were epilepsy, head trauma with loss of consciousness, former, or present substance abuse, suspicion of Autism spectrum disorder, prematurity (gestational age <36 weeks) or a full scale intelligence quotient (FSIQ) below 75, measured by the Wechsler Intelligence Scale for Children-IV (Wechsler, 2003). Among participants the following comorbid conditions were present at T2: oppositional defiant disorder (ADHD; *N* = 8, TS; *N* = 2), conduct disorder (ADHD; *N* = 1), phobia (ADHD; *N* = 5, TS; *N* = 3, control; *N* = 2), anxiety disorder (TS; *N* = 1), transient tics (ADHD; *N* = 2), chronic motor tics (ADHD; *N* = 1), obsessive compulsive disorder (TS; *N* = 3), depression (ADHD; *N* = 1, TS; *N* = 1) and elimination disorder (ADHD; *N* = 1). All children were medication-free and had no prior treatment for ADHD at the first assessment. Participants taking stimulants at the second assessment (21 participants, ADHD; *N* = 17, TS + ADHD = 4) were asked to refrain from taking the medication in the 48 h prior to testing. Other types of medication (antipsychotic 2nd generation + melatonin, TS; *N* = 1, antiepileptic, TS; *N* = 1) were taken as prescribed. Twenty-five children were girls and the groups did not differ for age and sex. Eleven children were left-handed (Table 1). The groups differed in FSIQ scores, similar to findings reported in other studies (Bornstein, 1991; Ozonoff et al., 1998; Baym et al., 2008; Debes et al., 2011). Groups did also differ in ADHD-RS total values. Total current tic severity in the TS group at T1 was 19.76 ± 8.71(range 4–38) and decreased significantly over time [T2: 14.47 ± 8.18, range 4–33, *t*_(1, 16)_ = 2.33, *p* = 0.03]. Lifetime worst-ever tic severity ranged from 14 to 23 (26.82 ± 8.13) at T1 and from 17 to 48 (31.00 ± 8.25) at T2, indicating a slight increase in the interval, consistent with the expected development (Leckman et al., 1998). The execution of many tics per day may trigger compensatory phenomena, but the fact that tics wax and wane in their frequency and characteristics over hours, days, and months, makes the objective measurement of symptom severity overall difficult. We therefore decided to use worst-ever tic severity for correlation with ERP measures as these might better relate to the accumulated symptom load and may represent a measure for compensatory long-term effects. To control for sampling bias, we compared the baseline characteristics from the first assessment between the dropouts (*N* = 29) and the returning participants (*N* = 70). Age at first examination, gender ratio or handedness did not differ across groups. However, a difference of FSIQ scores for the control group (96.8 at first assessment vs. 107.7 at second assessment) was found. This was not present in the two other groups (Table 1), indicating that the controls participating at T2 were biased toward a higher FSIQ. Sample characteristics, e.g., the ADHD-RS scores and the YGTSS scores did not differ across groups. Thus, despite some attrition, the present sample still was largely representative of the original samples, especially in the two diagnostic groups.

**Table 1:** Sample characteristics.

	**Controls *N* = 29**	**ADHD *N* = 24**	**TS *N* = 17**	**Statistics**
	**Mean ± *SD***	**Mean ± *SD***	**Mean ± *SD***	
**SAMPLE CHARACTERISTICS**				
Sex (% male)	58%	70%	65%	χ^2^ = 0.85, n.s.
FSIQ	107.69 ± 10.82	92.54 ± 8.09	99.00 ± 11.57	*F*_(2, 67)_ = 14.78, *p* < 0.001, $\eta_{p}^{2}$ = 0.31
**AGE (YEARS)**				
T1	10.12 ± 0.99	10.02 ± 1.28	9.84 ± 1.28	*F*_(2, 67)_ = 0.31, n.s.
T2	14.68 ± 1.15	14.49 ± 1.45	14.17 ± 1.79	*F*_(2, 67)_ = 0.69, n.s.
Handedness (% right handed)	93%	79%	76%	χ^2^ = 2.96, n.s.
**ADHD-RS TOTAL SCORE**				
T1	3.14 ± 2.97	30.54 ± 8.71	20.59 ± 9.98	*F*_(2, 67)_ = 95.18, *p* < 0.001, $\eta_{p}^{2}$ = 0.74
T2	4.81 ± 5.78	27.36 ± 10.19	19.39 ± 10.49	*F*_(2, 67)_ = 45.62, *p* < 0.001, $\eta_{p}^{2}$ = 0.58
**YGTSS TOTAL SCORE**				
**T1**				
Total current tic severity			19.76 ± 8.71	
Total worst-ever tic severity			26.82 ± 8.13	
**T2**				
Total current tic severity			14.47 ± 8.18	
Total worst-ever tic severity			31.00 ± 8.25	

*ADHD, Attention-Deficit/Hyperactivity Disorder; TS, Tourette Syndrome; FSIQ, full scale intelligence quotient; ADHD-RS, attention-deficit/hyperactivity disorder rating scale; SD, standard deviation; YGTSS, Yale Global Tic Severity Scale; T1, first assessment; T2, second assessment*.

### Experimental design

At both sessions electroencephalogram (EEG) was recorded during performance of a modified Eriksen flanker task implemented in the E-prime 2 experiment programming platform (Psychology Software Tools, https://www.pstnet.com/eprime.cfm) after verbal and written instruction and a training sequence. At the center of a PC screen, participants were presented a fixation dot. Trials began with the presentation of 6 horizontal flanker arrows appearing below the fixation. Participants were instructed to respond with their preferred hand as fast as possible and as accurate as possible with either a left or a right mouse button press following the direction of a central target arrow that appeared 100 ms after the flankers. The central target arrow pointed either into the same direction as the flanker arrows in compatible trials (<<< < <<<, >>> > >>>) or in the opposite direction in incompatible trials (<<< > <<<, >>> < >>>). The target and flanker-arrows remained on screen until a response was registered. Trials were terminated by a motor response and were followed by a fixed 800 ms interval before the onset of the next trial. Stimuli were presented in two blocks with 200 trials that were pseudo randomized separately for each participant. The overall probability of compatible and incompatible trials, as well as left and right responses was kept at 0.5, respectively. Performance feedback was given during the experiment when responses were erroneous or slower than the adaptive individual threshold value [mean response time plus 1.5 standard deviation (*SD*)].

### EEG data acquisition

EEG was recorded continuously in an electromagnetically shielded chamber (Rainford EMC Systems, Wigan, UK). Data were sampled at a 1,000 Hz-frequency with a 10 s time-constant, with Brain Amp MR plus X2 amplifiers (BrainProducts, Munich, Germany). An elastic cap containing 34 Ag/AgCl electrodes placed at Fp1, Fp2, F7, F3, Fz, F4, F8, FT9, FC5, FC1, FC2, FC6, FT10, T7, C3, Cz, C4, T8, TP9, CP5, CP1, CP2, CP6, TP10, P7, P3, Pz, P4, P8, PO9, O1, O2, PO10, Iz was used. Channels were recorded against Fz with a ground on Fcz. Vertical eye-movements were recorded with a bipolar derivation between Fp1 and an additional electrode placed below the left eye, horizontal eye movement were recorded with a bipolar derivation between Fp1 and Fp2. Additionally, electrocardiographic activity was monitored by an electrode placed on the left chest. Impedances were kept below 10 kΩ.

### EEG processing

Continuous EEG data files were imported into EEGLAB written in Matlab (Delorme and Makeig, 2004) and resampled to 500 Hz. The EEG-data were then re-referenced to a common average reference, and filtered from 0.1 to 40 Hz. After initial visual inspection to rule out focal or generalized EEG abnormalities, and pervasive non-stereotyped signal artifacts we performed automatic artifact rejection in order to denoise the data prior to spatial filtering with independent component analysis (ICA) in an unbiased way. The data were divided into 1000 ms epochs back-to-back. In order to derive a score with high sensitivity for artifacts these epochs were first detrended, and then we computed for each channel the absolute sums of the rectified epoch, as well as its differential. We also computed the standard deviation, skewness, and kurtosis of the time-series. The sum of the power spectrum was estimated, and we derived a dynamic range estimate by dividing the content at high frequencies by the low frequencies. These measures were normalized to unit variance, summed across all channels and normalized. Epochs within ±1 standard deviation were retained for further analysis, concatenated, and subjected to temporal ICA using infomax (Bell and Sejnowski, 1995). Thirty components were estimated after principal component analysis compression. The resulting component maps and activations were back-projected into the continuous data, and segmented into stimulus-locked (−0.5 to 1 s around flanker) and response-locked (−1 to 0.5 s around button press) sets. Hereafter, we automatically screened the components to retain only task and ERP-relevant sources. Firstly, spatial correlation with templates was used to find components relating to blinks and lateral eye movement (Viola et al., 2009). Then, spatially sparse components, i.e., loading only on single or few electrodes were identified by detecting outliers in the spatial standard deviation. In order to select ERP-relevant components, we first repeated artifact rejection as mentioned above, and, in addition discarded trials with response times <200 or >2,000 ms, and then generated component timecourses grand average stimulus and response-locked average waveforms; those components contributing variance to the overall ERP were kept (Wessel and Ullsperger, 2011).

### Averaging and data extraction

We generated ERP-averages from these back-projected data, separately for compatible, incompatible, and erroneous trials from both stimulus- and response-locked segments. We then inspected the grand averaged data across all participants together for both sessions to select regions and timewindows for statistical testing of relevant components. Based on our previous selection and upon inspection of grand average ERP data and difference waves across both age groups, we found that conditional effects on several components were consistently expressed around Cz/Vertex, which is in line with other work (Cycowicz, 2000; Stige et al., 2007). We therefore used a region average from FC1, FC2, Cz, CP1, CP2, for spatial data reduction, and controlling for inter-individual variability (Handy, 2005). Spatial averaging also helps to control for variability as seen in different age groups (e.g., Cycowicz, 2000; Davies et al., 2004; Brydges et al., 2013).

Because latency jitter in event-related potential components between trials, especially in children, and peak amplitudes can be influenced by group differences in signal-noise-ratio, analyses of mean amplitudes were chosen (Luck, 2005). Amplitudes were extracted from 40 ms long windows centered on the grand average peak latency and were used for testing of group differences. The time-windows we focused on for further analyses are the stimulus-locked P2 (170–210 ms), early P3 (310–350 ms), and late P3 (500–600 ms). In the response-locked averages, we extracted the ERN as the difference between −40 to 0 ms and 60 to 100 ms post-response. Pe was estimated as the average between 250 and 290 ms. For reference, we also included the earlier P2 component as a marker of exogenous processing. The stimulus-locked N2, detecting early stages of conflict/mismatch and is often reported in Flanker task studies on adults (Folstein and Van Petten, 2008), however, earlier studies revealed that adult-level N2 amplitudes were not found until the age of 16 (Ladouceur et al., 2007) and we decided therefore not to investigate this latency range further here in this age group.

### Statistics

To address our main question, we estimated the differences between groups, as well as effect modification with age and condition. This was done for the behavioral measures Accuracy (ACC), Reaction Time (RT), Reaction Time variability (sdRT), as well as the stimulus-locked ERPs P2, early P3, late P3, and response-locked ERN and Pe. We used a mixed-model analysis with repeated measures with an unstructured variance component matrix. The model included the within-subjects factor flanker outcome with three levels (compatible, incompatible, error) for RTs, with two levels (compatible, incompatible) for ACC, sdRT, and stimulus-locked ERP components, and with one level (error) for response-locked ERPs. In the longitudinal analysis, we included age at first and second assessment, as well as the three groups (Control, ADHD, TS) as between-subject factor. In the cross-sectional analyses, age was included as a covariate. Additionally, RT mean from both assessments was included as a within-subject factor. A random effect variable, assumed normally distributed, accounted for individual responses of the participants. The model was adjusted for all two-way interactions between the variables diagnostic group, age, and condition. We transformed Accuracy to Arcsine to ensure a normal distribution in the outcome measure. We considered *P* < 0.05 as statistically significant. *F*-tests were used for hypothesis testing on type three fixed effects and estimates are presented with 95% confidence intervals. All statistical analyses were conducted in R (R Development Core Team, 2008). Further, to investigate associations between neurophysiological data and tic scores, Pearson's correlations were computed in Statistica (Statsoft, Tulsa, OK, USA).

## Results

### Behavioral performance

**Figure 1:**
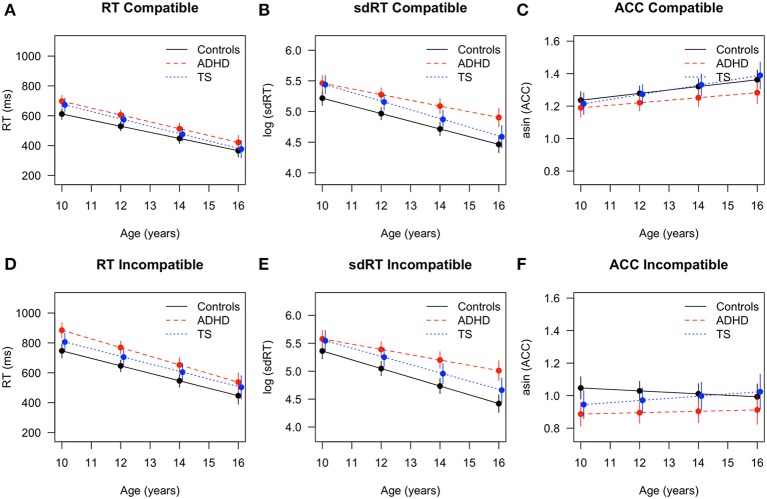
Flanker task performance with fitted values and 95% confidence intervals for children with TS (blue), children with ADHD (red), and control children (black). RT in **(A)** compatible and **(D)** incompatible trials, sdRT in **(B)** compatible and **(E)** incompatible trials, asin ACC in **(C)** compatible and **(F)** incompatible trials. ADHD, Attention-Deficit/Hyperactivity Disorder; TS, Tourette Syndrome; RT, reaction time; sdRT, standard deviation of reaction time; asin ACC, arcsine transformation of accuracy.

Reaction times decreased with age [*F*_(1, 371)_ = 744.06, *p* < 0.001] and showed group differences [*F*_(2, 67)_ = 6.17, *p* < 0.004, ADHD > TS > controls] (Figure 1). A typical Flanker effect (RT error < RT compatible < RT incompatible) was also present [*F*_(2, 335)_ = 153.13, *p* < 0.001]. An interaction of age by outcome approached significance [*F*_(2, 335)_ = 2.75, *p* = 0.06]. Directed *post-hoc* tests showed that, controls were faster than children with TS in compatible and incompatible responses at T1 (both RTs *p* < 0.05, erroneous RT *p* = 0.06). Developmental trajectories tended to converge for controls and children with TS during the second assessment where all reaction time differences were minimized (n.s., Figure 1). Controls responded faster than children with ADHD at the first assessment (all RT < 0.05), and those differences persisted in the second assessment (all RTs *p* < 0.05, except erroneous RT *p* = 0.06). Children with ADHD and with TS did not differ in terms of reaction times in either of the assessments.

Reaction time variability decreased with age [*F*_(1, 224)_ = 491.97, *p* < 0.001], and differed across conditions [*F*_(1, 201)_ = 11.67, *p* < 0.001, sdRT compatible < sdRT incompatible], without interaction [age × condition: *F*_(1, 201)_ = 2.14, n.s.]. Both diagnostic groups displayed a higher reaction time variability [*F*_(2, 67)_ = 8.67, *p* < 0.001, ADHD > TS > controls]. *Post-hoc* tests revealed that controls showed significantly lower variability both in compatible and incompatible trials than children with TS and ADHD at the first assessment (all sdRT *p* < 0.05), whereas children with TS and with ADHD did not differ. At the second assessment, differences between ADHD and controls persisted (all sdRT *p* < 0.01), while differences between children with TS and controls only were present in incompatible trials (*p* < 0.05). When comparing both diagnostic groups at T2, reaction time variability was lower in children with TS compared with ADHD in incompatible trials (*p* < 0.05), and slightly lower in compatible trials (*p* = 0.06). This pointed at diverging trajectories over time in both incompatible (Δ TS vs. Δ ADHD *p* < 0.01) and compatible trials (Δ TS vs. Δ ADHD *p* < 0.05) between these two groups. In addition, differences between children with ADHD and controls increased over time in incompatible trials (Δ controls vs. Δ ADHD *p* < 0.01).

Accuracy increased with age [*F*_(1, 225)_ = 14.88, *p* < 0.001], and differed across conditions [*F*_(1, 198)_ = 436.75, *p* < 0.001; ACC compatible > ACC incompatible; age × condition: *F*_(1, 198)_ = 10.33, *p* < 0.01]. Groups showed differences in accuracy across both conditions [*F*_(2, 64)_ = 3.31, *p* < 0.04, controls > TS > ADHD], without a clear interaction [group × condition: *F*_(2, 198)_ = 2.42, *p* < 0.09]. *Post-hoc* tests showed significant differences at the first assessment for incompatible trials between ADHD and controls (*p* < 0.01) and TS and controls (*p* < 0.05), but no group differences between TS and ADHD. Developmental trajectories converged at the second assessment for controls and children with TS and all differences in accuracy were minimized (n.s.). Differences between controls and ADHD persisted (*p* = 0.02), and children with TS were slightly more accurate than children with ADHD (*p* = 0.06, TS > controls > ADHD). In compatible trials, accuracy improved most in children with TS (Δ change *p* = 0.03), followed by controls (Δ change *p* = 0.05), while ADHD showed no relevant improvement. Performance for incompatible trials was more variable, and only children with TS showed a trend toward improved accuracy over time in incompatible trials (Δ change *p* = 0.08), while the other groups remained unchanged overall.

### Electrophysiological results

#### Response-locked components

The ERN amplitude became larger with age [*F*_(1, 90)_ = 95.14, *p* < 0.001] without an apparent main effect or interaction of other terms [group: *F*_(2, 66)_ = 1.54, n.s.; age × group: *F*_(2, 91)_ = 2.15, *p* = n.s.]. Direct comparison of longitudinal changes showed that ERN amplitudes increased from the first to the second assessment for all groups, however, average ERN amplitude appeared to become larger for controls and children with TS compared to children with ADHD, though without reaching significance (Δ change controls and Δ change TS: *p* < 0.01, Δ change ADHD *p* < 0.07).

**Figure 2:**
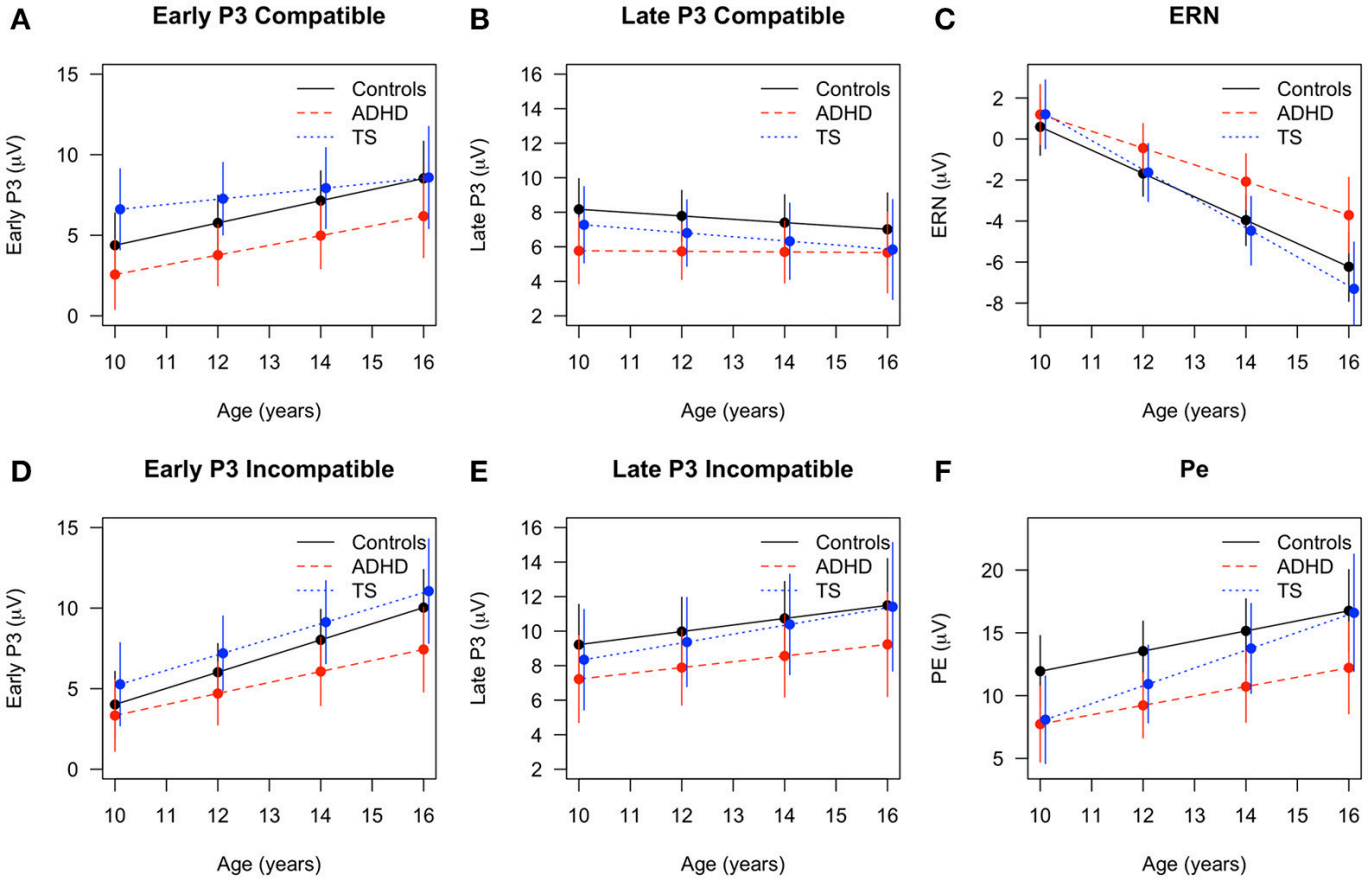
Developmental trajectories across age in event-related components with fitted values and 95% confidence intervals for children with TS (blue), children with ADHD (red), and control children (black). Early P3 in **(A)** compatible and **(D)** incompatible trials, late P3 in **(B)** compatible and **(E)** incompatible trials, **(C)** error-related negativity (ERN) in erroneous trials, **(F)** Pe in erroneous trials.

**Figure 3:**
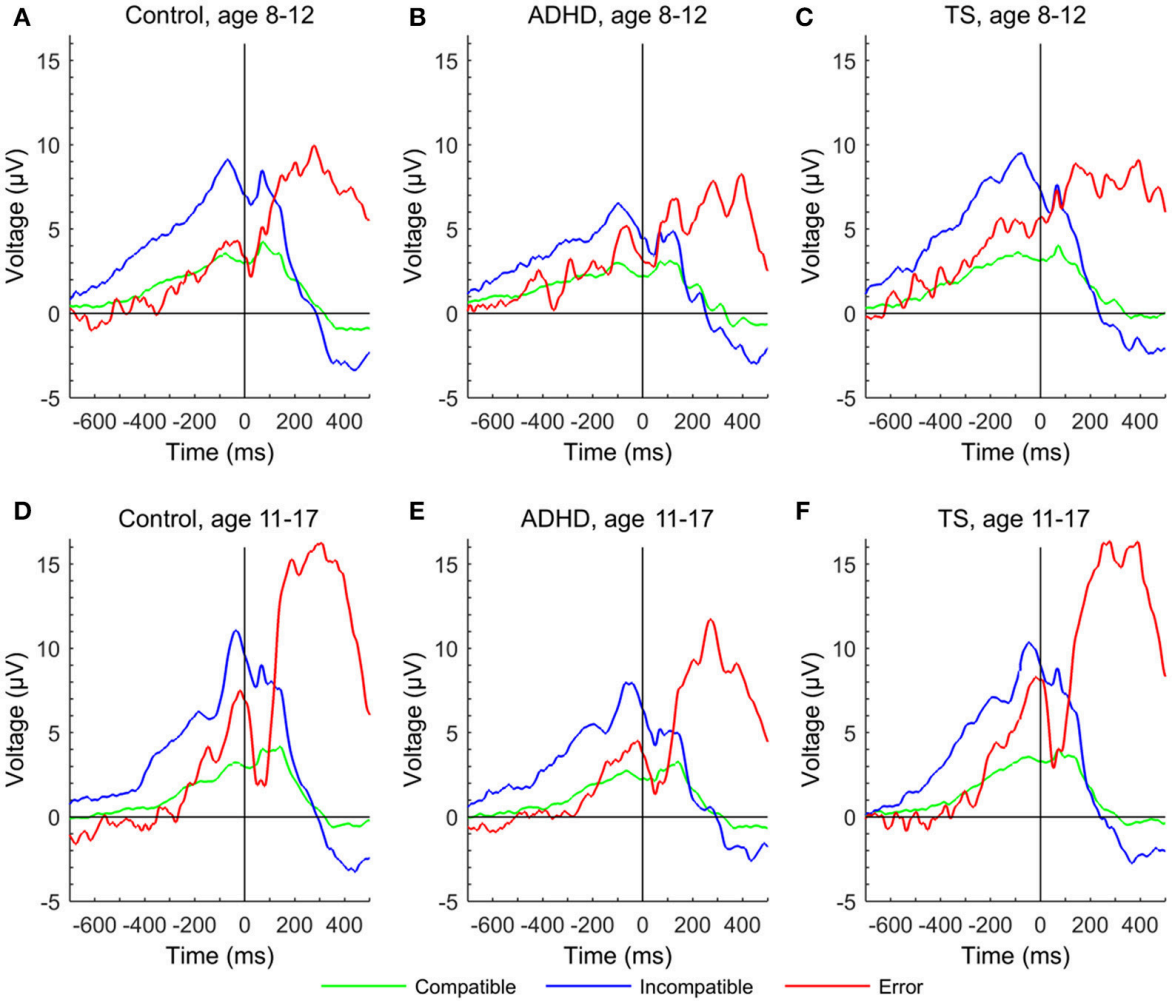
Error-related negativity and Pe in response-locked event-related potentials at a central region of interest to compatible (green), incompatible (blue), and erroneous trials (red) of the Flanker task for each group at first and second assessment. **(A)** control group at age 8–12 years, **(D)** control group at age 11–17 years, **(B)** ADHD group aged 8–12 years, **(E)** ADHD group aged 11–17 years, **(C)** TS group at age 8–12 years, **(F)** TS group at age 11–17 years.

Pe amplitude also increased with age [*F*_(1, 84)_ = 23.46, *p* < 0.001], and simultaneously showed an inverse relation with response time changes [*F*_(1, 131)_ = 7.45, *p* < 0.01]. Amplitudes differed across groups [*F*_(2, 67)_ = 3.16, *p* < 0.05] across both assessments with ADHD children showing lowest levels, and no interaction of age with group [*F*_(2, 84)_ = 1.18, n.s.]. Although children with TS appeared on average to show a slightly higher change over time than children with ADHD, no significant *post-hoc* group differences were found (Figures 2, 3).

#### Stimulus-locked components

Younger participants had a higher P2 amplitude than older ones [*F*_(1, 222)_ = 17.94, *p* < 0.001]. Groups overall displayed similar P2 amplitudes [*F*_(2, 66)_ = 0.81, n.s.], meaningful interactions were not seen, and developmental trajectories showed no relevant differences across groups.

The early P3 showed a significant increase over time [*F*_(1, 223)_ = 71.22, *p* < 0.001], also showing an inverse relation with response time [*F*_(1, 265)_ = 5.70, *p* < 0.05]. The overall developmental change did not differ between the groups in the full model [age × group: *F*_(2, 215)_ = 1.16, n.s.], but *post-hoc* tests showed that children with TS had larger amplitudes in compatible trials than children with ADHD (*p* = 0.01) at the first assessment. Over time, compatible P3 increased for controls and children with ADHD (Δ change controls *p* < 0.01, Δ change ADHD *p* = 0.07, Δ change TS n.s.). In incompatible trials, only controls increased in amplitude (Δ change control: *p* = 0.01, Δ change TS and ADHD n.s.).

**Figure 4:**
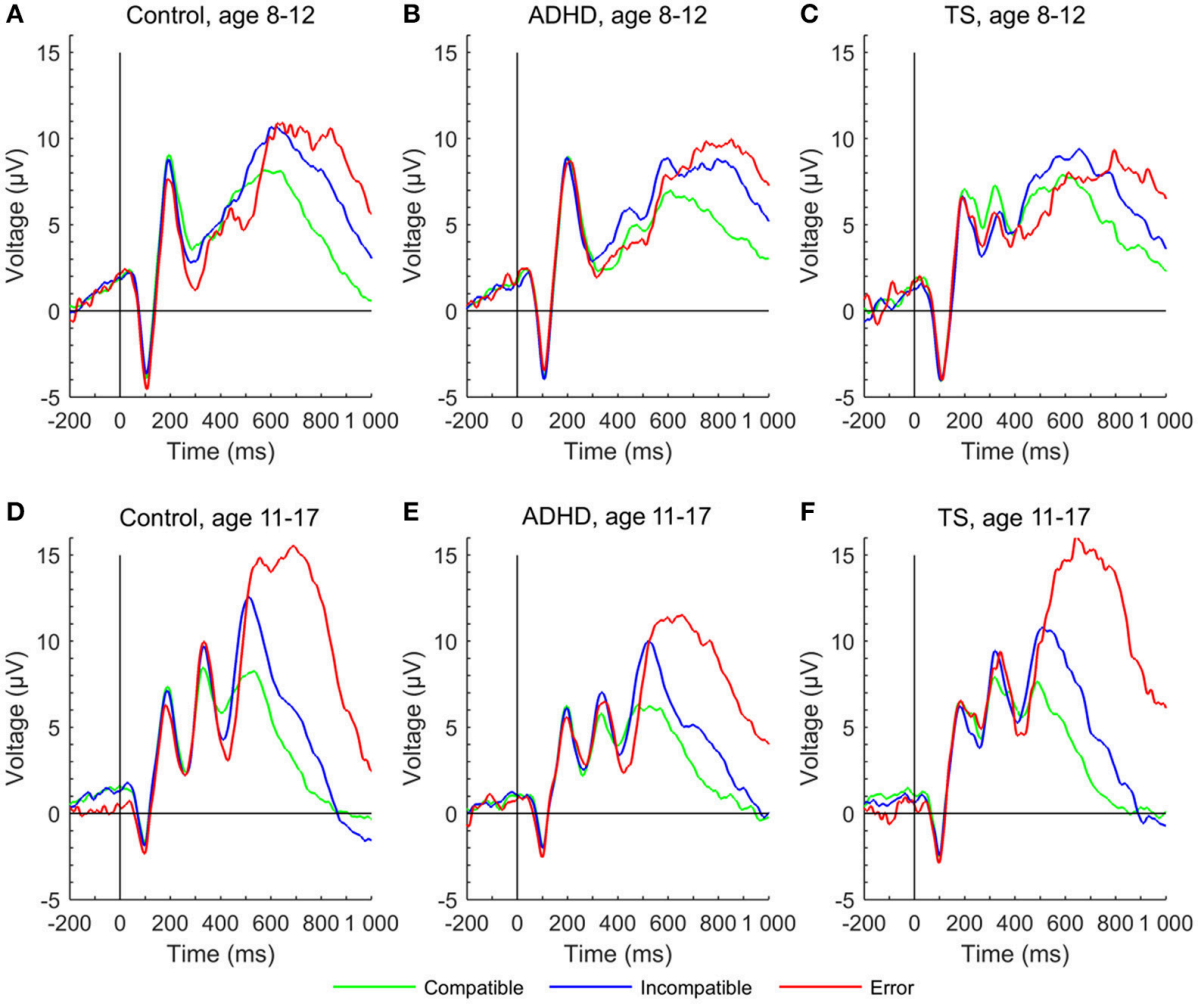
P2, early P3, and late P3 in stimulus-locked event-related potentials at a central region of interest to compatible (green), incompatible (blue) and erroneous trials (red) of the Flanker task for each group at first and second assessment. **(A)** control group at age 8–12 years, **(D)** control group at age 11–17 years, **(B)** ADHD group aged 8–12 years, **(E)** ADHD group aged 11–17 years, **(C)** TS group at age 8–12 years, **(F)** TS group at age 11–17 years.

The late P3 differed between conditions [*F*_(1, 200)_ = 27.25, *p* < 0.001], with larger amplitudes in incompatible trials and an inverse relation with response time [*F*_(1, 264)_ = 11.78, *p* < 0.001]. Late P3 amplitudes in compatible trials tended to decrease, whereas incompatible amplitudes marginally increased over time [*F*_(1, 227)_ = 3.16, *p* = 0.07; age × condition: *F*_(1, 200)_ = 9.14, *p* < 0.01]. Diagnostic groups did not differ in their developmental trajectories [age × group: *F*_(2, 223)_ = 0.10, *p* = n.s.] (Figures 2, 4).

### Exploratory analyses

#### Correlations between ERP amplitudes and YGTSS scores

**Figure 5:**
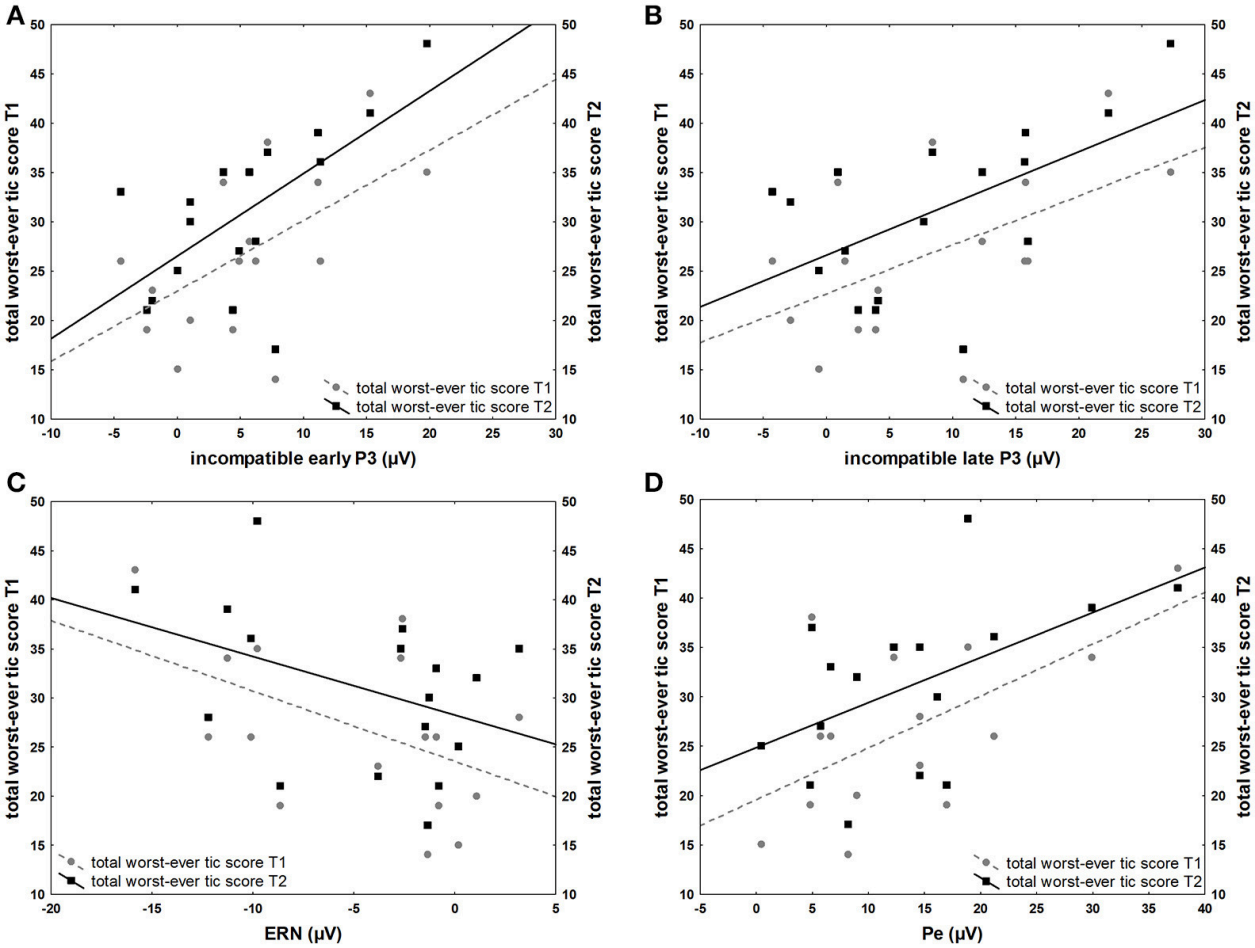
Associations between ERP amplitudes and YGTSS scores. **(A)** Positive correlation of total worst-ever tic severity (range 0–50) at first (T1) and second (T2) assessment and incompatible early P3 amplitude (μV) of the first assessment **(B)** Positive correlation of total worst-ever tic severity at first (T1) and second (T2) assessment and incompatible late P3 of the first assessment **(C)** Inverse correlation of total worst-ever tic severity at first (T1) and second (T2) assessment and ERN the second assessment **(D)** Positive correlation of total worst-ever tic severity at first (T1) and second (T2) assessment and Pe of the second assessment.

Children with TS showed significant positive correlations between worst-ever total tic scores collected at the first assessment and stimulus-locked amplitudes for the first assessment, compatible P2 (*r* = 0.61, *p* < 0.01), incompatible P2 (*r* = 0.67, *p* < 0.01), compatible early P3 (*r* = 0.50, *p* = 0.04), incompatible early P3 (*r* = 0.57, *p* = 0.02), incompatible late P3 (*r* = 0.54, *p* = 0.02). The ERN, however, correlated inversely with worst-ever total tic scores (*r* = −0.49, *p* < 0.05) for the second assessment, where this component was developed in contrast to the first assessment, where no apparent ERN was present in most participants. A positive correlation was also present with Pe from the second assessment (*r* = 0.61, *p* < 0.01). Worst-ever tic scores from the second assessment correlated positively with stimulus-locked amplitudes from the second assessment, compatible P2 (*r* = 0.58, *p* = 0.02), incompatible P2 (*r* = 0.71, *p* < 0.001), compatible early P3 (*r* = 0.52, *p* = 0.03), incompatible early P3 (*r* = 0.65, *p* < 0.01), incompatible late P3 (*r* = 0.56, *p* = 0.02). A positive correlation was present for the Pe from the second assessment (*r* = 0.53, *p* = 0.04) (Figure 5). Current total tic severity measures did not significantly correlate with YGTSS measures.

#### Influence of comorbid ADHD in children with TS

To ensure the adequacy of treating children with TS with and without ADHD as one group, we also conducted the mixed-model analysis for the main behavioral and ERP results with four groups (TS only, TS+ADHD, controls, and ADHD), and summarized overall test results here, along with directed *post-hoc* tests between TS only and TS+ADHD.

For RT, the analysis showed group differences as previously [*F*_(3, 65)_ = 4.00, *p* = 0.01]. *Post-hoc* tests between TS only and TS+ADHD were not significant for all RTs at both assessments (all RT *p* > 0.32). Developmental trajectories did not differ between those two subgroups (all Δ change *p* > 0.19).

Reaction time variability yielded a group main effect of similar strength [*F*_(3, 65)_ = 5.53, *p* = 0.001]. All *post-hoc* tests at both assessments between TS only and TS+ADHD were not significant (all sdRT *p* > 0.20). However, trajectories diverged between TS and TS+ADHD in compatible trials (Δ change *p* = 0.007) with lower reaction time variabilities for the TS only group over time, but not in incompatible trials (Δ change *p* = 0.08).

Groups showed differences in accuracy across both conditions [*F*_(3, 63)_ = 2.17, *p* = 0.01]. *Post-hoc* tests for both assessments as well as developmental trajectories did not differ between TS only and TS+ADHD (all ACC *p* > 0.84, all Δ change *p* > 0.79).

For the response-locked ERN, no significant group differences were found in the analysis with four groups. As noted previously, ERN was not developed in at T1 across groups, and no significant differences between subgroups were seen at T2 (*p* > 0.55), or over time (Δ change *p* = 0.32).

For the Pe, the analysis with four groups repeats a group difference [*F*_(3, 66)_ = 2.70, *p* = 0.05], and shows a trend-significant *post-hoc* test at T1 (*p* = 0.07, TS+ADHD > TS), however trajectories merged between the groups and no differences were found at T2 (*p* > 0.82, Δ change *p* = 0.16).

The stimulus-locked P2 remained without differences between groups overall, component estimates and their changes over time were similar for TS only and TS+ADHD (all *p* > 0.13, all Δ change *p* > 0.23).

Similarly, early P3 results from the main analysis were repeated, no *post-hoc* groups differences between TS only and TS+ADHD were seen (all *p* > 0.53, all Δ change *p* > 0.83).

The overall developmental change of the late P3 revealed at trend-significant difference between the groups in the full model [age × group: *F*_(3, 220)_ = 2.57, *p* = 0.06], but *post-hoc* tests did not show differences between the two subgroups at T1 and T2 (all *p* > 0.12). However, incompatible late P3 amplitudes increased more over time in the TS only group compared to TS+ADHD, resulting in different trajectories for incompatible trials (Δ change *p* = 0.04), but not for compatible trials (Δ change *p* = 0.18).

While it is noteworthy that some of the measures here showed differences between TS subgroups at T1 or T2, or in terms of change over time, the majority of the data available to us showed no sign of more widespread differences when using two-sample *t*-tests and effect size estimators.

## Discussion

The present examination of attention, cognitive control, and performance monitoring from childhood to adolescence revealed several differences of the developmental trajectories between children with TS, and healthy controls, and in contrast to children with ADHD. Task performance in terms of reaction times, reaction time variability, and accuracy improved in all groups with age, but behavioral differences between children with TS and controls diminished at the second assessment, whereas differences between controls and children with ADHD largely persisted. In terms of ERP, the early P3 developed earlier in children with TS compared with controls and trajectories converged with maturation. Worst-ever total tic scores correlated positively with stimulus-locked ERP from the first and inversely with response-locked ERP from the second assessment in children with TS.

The developmental effects on performance for the overall group with increased accuracy, faster reaction times, and decreased reaction time variability are consistent with the literature (Posner and Rothbart, 2007; Tamnes et al., 2013). Children with TS improved in most performance measures over time, resulting in converging trajectories between controls and TS following an initial deficit at T1 in children with TS, in terms of reaction times, reaction time variability and accuracy. Compared with children with ADHD, children with TS showed bigger reductions in their reaction time variability, resulting in significant differences between both groups at the second assessment. When additionally separating the two subgroups with and without ADHD comorbidity we saw that reduction of variability was more prominent in the TS only group. The adaptation of behavioral performance in children with TS is a topic of discussion and existing studies report inconsistent results (Harris et al., 1995; Serrien et al., 2005; Mueller et al., 2006; Jackson et al., 2007; Baym et al., 2008; Roessner et al., 2008; Eichele et al., 2010; Shephard et al., 2016a). Only a few other, behavioral, studies have used variants of flanker tasks in children with TS and found that TS is not associated with widespread executive impairment, however children with comorbid conditions tended to perform less well (Ozonoff et al., 1998; Crawford et al., 2005). While most of our measures show no significant differences between subgroups in this sample, the presence of increased response time variability in comorbid ADHD is not unexpected due to the high penetrance of this feature in ADHD (Klein et al., 2006). However, most studies included participants with a wider age range, or an age range consistent with our first assessment. To our knowledge, no other study assessed behavioral changes over time in children in Tourette syndrome. Differences between first and second assessment in our study may point to adaptation of compensatory self-regulation mechanisms from childhood to adolescence through the constant need to suppress tics. Tic suppression may lead to increased control over motor outputs and by that generalizes to behavioral measures of cognitive control (Mueller et al., 2006; Jackson et al., 2015). On the other hand, our results with differences in the early P3 possibly point at an earlier implementation of adaptive effects already during stimulus evaluation, at least in the context of this task (Eichele et al., 2016) and our exploratory analyses of ERP amplitudes with tic severity further seem to support this notion.

In contradistinction, despite general improvement in performance over time in the ADHD group, most of the ADHD-related performance differences with respect to behavioral performance persisted through development in direct comparison with the controls. The significant group-effect for reaction time variability and error rate in children with ADHD is in line with findings that increased variability and error rate are particularly robust markers of ADHD (for review, see Mullane et al., 2009; Kofler et al., 2013; Michelini et al., 2016). Impairments in those behavioral measures are thought to result from lapses in attention in the flanker congruent condition and as failure in executive control in the incongruent condition (Michelini et al., 2016). This may point to a pattern of performance deficits consistent with the developmental lag model (Doehnert et al., 2010) and probably in relation to reduced activity in the anterior cingulate cortex, and the functional networks in which it is involved (Plessen et al., 2016).

Maturation had a strong effect on the ERP components in the overall group with younger children showing smaller amplitudes in ERN, PE, early P3, and incompatible late P3 while P2 and compatible late P3 amplitudes decreased with older age. The age effects on the error-related components for the overall group with increasing amplitudes of ERN through adolescence are consistent with prior studies (Davies et al., 2004; Ferdinand and Kray, 2014). We see a similar, but weaker maturation effect for the Pe independent of group. However, this change in amplitude was not present in the respective data reported by Davies et al. (2004) and Wiersema et al. (2007), whereas, a clear difference was present in the grand average waveforms presented by Ladouceur et al. (2004). With respect to the diagnostic groups, children with ADHD showed an attenuated increase in ERN amplitude from the initial to the second assessment in contrast to the children with TS and controls. Groups did not show different developmental trajectories of the Pe. This is in line with a recent meta-analysis comparing ADHD with controls and reporting an overall attenuation of ERN in performance-monitoring tasks while Pe attenuations were not significant in Flanker tasks for the ADHD group (Geburek et al., 2013). This attenuation of the typical increase in ERN amplitude over a period of 4.5 years confirms a deficit of the early detection of an error, whereas the following stage of error processing appears to be less affected in juvenile ADHD. Remarkably, albeit not significant, the gradient of increase in Pe amplitude was steepest in TS (Figure 3F), leading to reduction of group differences between controls and TS while trajectories of children with ADHD was flatter.

Larger amplitudes in early P3 in children with TS compared with controls were seen in the first assessment (Eichele et al., 2016), and interpreted as a reflection of sustained effort in the TS group to intensify stimulus processing with an increased focused attention to the stimuli, leading to an altered target discrimination pattern. Over time, developmental trajectories of the compatible early P3 amplitude in control children appear to catch up with the TS group, merging in the second assessment on a similar level, whereas at the same time ADHD children trailed both other groups. Remarkably, children with TS maintain the largest amplitudes in incompatible trials at T2, with a very similar increase over time compared with controls, while the trajectory for the children with ADHD flattened, yielding a slightly increased amplitude difference. The deviant P3 trajectory for the children with ADHD thus persisted into mid-adolescence, despite effects of maturation in all groups. Additional studies are needed to characterize the functional role of this P3 subcomponent in TS in the context of change detection/oddball designs as well as Go/Nogo-type experiments.

For the remaining stimulus-locked ERP amplitudes, we observed marginal changes over time. Across trial conditions, ADHD showed smallest amplitudes in the late P3, while TS and controls showed larger amplitudes. There was no appreciable change of compatible amplitudes for the ADHD group, while the TS and control groups showed subtle amplitude reduction over time. On the other hand, the developmental trajectories of the incompatible late P3 increased slightly, and merged in adolescence for TS and controls, but not for the ADHD group. This suggests normal maturation of attention functions and non-significant attenuation in children with TS. For the P2 we expected an amplitude reduction over time based on normative studies (Allison et al., 1983; Mahajan and McArthur, 2012) on visual evoked responses, and this was the case across the entire sample.

The moderate to strong correlations between several ERP amplitudes and the worst-ever tic scores are striking. Positive correlations with stimulus-locked ERP from the first and inversely with response-locked ERP from the second assessment may suggest that the associations provide an indicator for a higher symptom load earlier in life leading to changes in stimulus and motor processing, resulting in a an inverse correlation with error-related amplitudes at a later stage. It has been proposed that children with TS gain tic control through compensatory mechanisms that involve alteration of prefrontal control over motor output, experimental support comes specifically from supplementary motor area (Jackson et al., 2015). Consistent with this, transcranial magnetic stimulation studies have demonstrated that the pre-supplementary motor area may modulate primary motor cortex activity in conflicting situations and thus influence corticospinal excitability (Mars et al., 2009). We suggest that such functional adaptations more generally may affect cognitive control feedback loops, i.e., extending to the functional systems in the medial frontal wall including the cingulate gyrus (Ullsperger et al., 2014). In particular, cingulate cortex activity predicts motor cortex activity, and changes in behavior, as well as subsequent activity in sensory cortices on a trial-by-trial basis, presumably in order to provide optimal performance (Danielmeier et al., 2011). It is well-established that the main generator of the ERN/Pe is located in mesial frontal cortex (Debener et al., 2005), therefore an inverse relation between ERN and tic severity, intuitively, could be an indicator of such a functional adaptation. This impression is further supported by the fact that some generators of P3 sub-processes that represent attentional control are located in the medial frontal cortex (Gehring and Fencsik, 2001; Huster et al., 2013). Surprisingly, correlations were also present with P2. However, we assume that P2 represents primarily sensory processing such that an indirect effect of medial frontal control seems plausible explanation at this point (Danielmeier et al., 2011, 2015). The current analysis was not set up to specifically investigate trial-to-trial connectivity, and concurrent behavior, but ongoing work in our lab further investigates these correlations with time-frequency analysis.

Among the limitations of our study, the relatively small sample size should be mentioned which also required disregarding comorbidities, use of medication, as well as the utilization and efficacy of behavioral treatment options. The latter information was unfortunately not available within the current study setup. Ideally, the impact of comorbid conditions and medication should be assessed separately, and in more detail, however, we performed explorative *t*-tests between these subsamples in the dependent measures and did not find any significant differences between these subsamples. To complement the t-statistics, we also estimated Hedges g for the total TS sample vs. ADHD, and for the TS-ADHD subgroup vs. ADHD from all relevant dependent measures, and found no robust differences in terms of average effect size. This may justify the inclusion of children with TS only and those with additional ADHD in the same group. The relative lack of negative impact of comorbid ADHD on TS in our sample seems at variance with previous work reporting impaired ERPs (Shephard et al., 2016a) and behavior (Roessner et al., 2007; Sukhodolsky et al., 2010; Greimel et al., 2011; Shephard et al., 2016a) in participants with TS and ADHD. However, differences in mean age and gender distribution of the sample, as well as use of medication are different. Differences in task design and time on task may also play a role. Further, we lost several participants to follow-up yielding sample attrition, which could have altered the sample, especially in the TS group. We conducted *t*-tests of the baseline characteristics between participants lost to follow-up and remaining participants, both for the overall group as well as for the diagnostic groups separately and found no significant differences. We also reviewed the reasons for dropout based on debriefing information for the TS group, only one child did not further participate due to reduction of symptoms, for the remaining seven participants that did not return, other or no reasons were given.

## Conclusion

Taken together, the present examination of cognitive processes from childhood to adolescence helps us to further broaden our knowledge of electrophysiological correlates in children with TS over time. During development, electrophysiological and behavioral differences between controls and children with TS decreased and trajectories converged with control children. This may point at compensatory adaptive processes that mitigate symptoms load in children with TS over time. This is in contradistinction to residual deficits in adolescents with ADHD which continued to show reduced performance at both time points. However, some reduction of deficits in absolute terms was also apparent for other measures upon visual inspection, and may have missed significance due to lower statistical power with our overall small sample size.

The developmental changes seen in children with TS support theoretical accounts of the development of cognitive control stating that the ongoing maturation of the prefrontal circuitries, including the ACC, plays a major role in the development of cognitive control (Posner and Rothbart, 2007; van Meel et al., 2012). Further, our findings may support the assumption of the frontal compensatory self-regulation hypothesis linking control over tics with an adaptation of functions in the midfrontal cortex, and its control over motor output already in childhood.

The here presented indications of early compensatory effects may contribute to clinical awareness concerning underlying neurobiology of plastic processes and compensatory effects already in earlier age that may indicate that children with TS could benefit from habit reversal training already before the recommended age. Further, enhancements in adaptive processes through specific interventions may in the future decrease some of the impairments associated with TS, and thus improve quality of life in this patient group.

## Author contributions

Conception and design: HE, TE, LS, KH, and KP. Acquisition of data: HE, TE, LM, SA, and LS. Analysis and interpretation, writing of article: HE, TE, and KP. Critical review of article and agreement to be accountable for all aspects of the work: HE, TE, LM, SA, KH, LS, and KP.

### Conflict of interest statement

The authors declare that the research was conducted in the absence of any commercial or financial relationships that could be construed as a potential conflict of interest. The reviewer MB and handling Editor declared their shared affiliation, and the handling Editor states that the process nevertheless met the standards of a fair and objective review.
